# Feasibility of opportunistic bone health assessment using low-dose CT trabecular attenuation and ^18^F-NaF uptake from PET/CT in prostate cancer

**DOI:** 10.3389/fendo.2026.1865099

**Published:** 2026-07-20

**Authors:** Cailing Liu, Fenghua Liang, Zuguo Li, Zhenzhen Wang, Wei Fu, Xingyu Mu

**Affiliations:** Department of Nuclear Medicine, The First Affiliated Hospital of Guilin Medical University, Guilin, China

**Keywords:** ^18^F-NaF PET/CT, bone health, opportunistic screening, osteoporosis, prostate cancer, trabecular attenuation

## Abstract

**Background:**

^18^F-NaF PET/CT is widely used in prostate cancer for detecting skeletal metastases, but the examination also contains structural and metabolic information potentially relevant to bone health assessment.

**Objective:**

This study evaluated feasibility of this dual-parameter approach for opportunistic bone health assessment in prostate cancer.

**Design:**

This was a retrospective cross-sectional.

**Methods:**

A total of 105 men with prostate cancer without bone metastasis underwent ^18^F-NaF PET/CT. Stand-alone diagnostic CT-L1 was available in 76 patients and was used as an external structural reference for feasibility analysis and gray-zone stratification. Patient-level analyses used mean lumbar SUV_avg_ and mean PET/CT-derived trabecular attenuation across L1-L4. Vertebra-level analyses used all available same-level SUV_avg_ and same-level PET/CT-derived trabecular attenuation measurements. Patient-level metabolic-structural discordance on ^18^F-NaF PET/CT was assessed.

**Results:**

PET/CT-derived L1 trabecular attenuation agreed strongly with stand-alone diagnostic CT-L1, with a correlation coefficient of 0.929 and an intraclass correlation coefficient of 0.916. In the paired diagnostic CT, cross-validated discrimination for low diagnostic CT-L1 thresholds was already high with low-dose CT alone, and adding SUV did not improve performance. However, among 41 patients in the stand-alone diagnostic CT gray zone of 110–160 HU, 19 (46.3%) were classified as low ^18^F-NaF uptake. At the patient level, mean SUV_avg_ across L1-L4 correlated positively with mean PET/CT-derived trabecular attenuation across L1-L4 (r = 0.521, p<0.001). In the vertebra-level mixed-effects model, each 10-HU increase in trabecular attenuation was associated with a 0.084-unit increase in SUV_avg_ (95% CI: 0.058 to 0.110; p<0.001). Nine patients showed a higher-TA but low-uptake phenotype, whereas 30 showed low TA with higher uptake.

**Conclusions:**

In prostate cancer, ^18^F-NaF PET/CT may feasibly support opportunistic bone health assessment by integrating low-dose CT-derived trabecular attenuation and ^18^F-NaF uptake within the same examination. The clinical contribution of ^18^F-NaF uptake appears to lie more in refining gray-zone stratification and identifying metabolically discordant patients. These feasibility findings are hypothesis-generating and require prospective validation against DXA and fracture endpoints before clinical use.

## Introduction

Prostate cancer (PCa) predominantly affects older men, a population already vulnerable to age-related bone loss. This background risk is often amplified by treatment, particularly androgen deprivation therapy (ADT), which accelerates bone loss and increases the risk of fragility fracture (1–4). Fractures are associated with substantial morbidity and mortality, and current guidance recommends baseline bone health assessment with dual-energy X-ray absorptiometry (DXA) in men starting ADT (1–4). In routine practice, however, implementation of bone health assessment remains inconsistent, and population-based studies have shown low rates of DXA or broader bone health monitoring after ADT initiation (5, 6). There is therefore a clear need for pragmatic opportunistic screening strategies that leverage imaging already being performed for oncologic care, without additional radiation exposure, extra visits, or separate testing.

Measurement of vertebral trabecular attenuation (TA) in Hounsfield units (HU) on routine computed tomography (CT) has emerged as a practical opportunistic screening approach. Lumbar TA, particularly at L1, correlates with DXA-derived bone mineral density and provides a pragmatic structural surrogate of skeletal fragility (7–11). This concept has also been extended to oncologic imaging cohorts, including prostate cancer PET/CT populations (12, 13). However, TA remains a structural measure. Although very low HU values strongly suggest low bone density, many patients fall into an intermediate range, commonly around 110–160 HU, in which structural attenuation alone may not adequately discriminate risk (7, 10, 14). This gray zone is clinically relevant because biologic deterioration in bone remodeling may precede overt structural loss.

^18^F-sodium fluoride (^18^F-NaF) positron emission tomography/computed tomography (PET/CT) is well established in prostate cancer for the sensitive detection of osseous metastases (15–17). Yet this hybrid examination inherently combines a low-dose CT component with a bone-seeking metabolic tracer. Beyond focal lesion detection, ^18^F-NaF uptake reflects regional perfusion, hydroxyapatite binding, and osteoblastic remodeling (16, 17). In our previous DXA-anchored study, lower lumbar ^18^F-NaF uptake, quantified as mean standardized uptake value (SUV_avg_), was strongly associated with lower bone mineral density, and pragmatic uptake thresholds were derived for translational use (18). These observations raise the possibility that ^18^F-NaF PET/CT could function as a dual-parameter platform for opportunistic bone health assessment by integrating structural TA and metabolic uptake within the same examination.

Despite this biologic rationale, the use of ^18^F-NaF PET/CT as an integrated opportunistic bone health tool in prostate cancer remains largely unexplored. We therefore aimed to evaluate a dual-parameter framework in men with PCa by assessing the structural feasibility of the low-dose CT component, the incremental value of ^18^F-NaF uptake for gray-zone stratification, the association between ^18^F-NaF uptake and TA at patient and vertebral levels, and the presence of metabolic-structural discordance within the same examination.

## Materials and methods

### Study design and population

This retrospective cross-sectional study was reported in accordance with the Strengthening the Reporting of Observational Studies in Epidemiology (STROBE) statement (19). Eligible patients were adult men with pathologically confirmed prostate cancer who underwent ^18^F-NaF PET/CT at our institution between September 2021 and July 2025. Exclusion criteria were: (1) confirmed skeletal metastases; (2) incomplete imaging data or image quality insufficient for reliable structural or metabolic assessment; and (3) documented synchronous metabolic bone disease.

Baseline demographic and clinical characteristics, including age, smoking status, indication for ^18^F-NaF PET/CT (initial staging or follow-up), and duration of previous treatment, were extracted from the medical record. The study was conducted in accordance with the Declaration of Helsinki. Given the retrospective analysis of anonymized data, the requirement for individual informed consent was waived by the Institutional Review Board (Approval Number: 2023QTLL-16).

### ^18^F-NaF PET/CT and CT acquisition

All patients underwent ^18^F-NaF PET/CT according to institutional clinical workflow, broadly consistent with European Association of Nuclear Medicine procedure recommendations for ^18^F-NaF bone imaging (20). After intravenous administration of ^18^F-NaF and an uptake period of approximately 60 min, imaging was performed from head to foot.

PET/CT acquisition was performed on a NeuWise PET/CT scanner (Neusoft, Shenyang, China). PET images were reconstructed using ordered-subset expectation maximization (25 iterations, 21 subsets; matrix 256 × 256). The low-dose CT component used for attenuation correction was acquired at 120 kV, 160 mAs, and 1-mm slice thickness.

Stand-alone diagnostic CT examinations were performed at our medical center using multidetector CT scanners from a single vendor (GE Healthcare, USA), including the Revolution ES, LightSpeed VCT, and Optima CT660 systems. Briefly, chest and/or abdominal CT examinations were performed by using a variety of multidetector CT scanners at a constant peak voltage of 120 kV with variable tube current values that were protocol specific but clinically appropriate for achieving a diagnostic examination for the given indication. Tube current was protocol- and scanner-dependent with automatic modulation, typically ranging from 40 to 200 mA on the Revolution ES, 200 to 500 mA on the LightSpeed VCT, and 50 to 600 mA on the Optima CT660. We included examinations either with or without intravenous and oral contrast agent (the use of intravenous contrast agent was recorded).

### Imaging-derived variables

**Figure 1:**
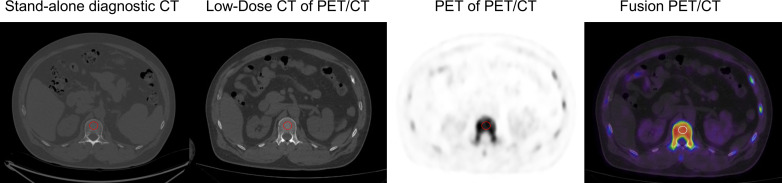
Example of region of interest.

Trabecular attenuation was measured on the low-dose CT component of ^18^F-NaF PET/CT at L1-L4 and on stand-alone diagnostic CT at L1. For each vertebra, TA was measured in HU on a single axial slice obtained parallel to the endplates at the pedicle level. A fixed 200-mm² elliptical region of interest was manually placed within the trabecular compartment in the anterior third of the vertebral body. Cortical bone, focal sclerotic or lytic changes, endplate sclerosis, and visible vascular channels were carefully avoided (**Figure 1**). This approach was aligned with previously validated opportunistic CT methods (7–11).

For metabolic assessment, ^18^F-NaF uptake was quantified as SUV_avg_. Because PET and CT were co-registered within the same examination, SUV_avg_ was extracted using ROIs matched to the CT-derived trabecular ROIs at the corresponding lumbar levels. Mean lumbar SUV_avg_ across L1-L4 was then calculated as the patient-level metabolic summary measure.

### Threshold definitions and cross-validated discrimination

Three binary reference outcomes were defined from stand-alone diagnostic CT-L1: <110 HU, <135 HU, and <160 HU, based on prior opportunistic CT literature (7, 10). These cut-points were prespecified and adopted directly from established opportunistic-CT studies; they were applied in an exploratory manner.

For each outcome, two logistic regression models were evaluated using PET/CT-derived L1 variables: a low-dose CT-only model including L1 PET/CT-derived TA alone, and a low-dose CT plus SUV model including L1 PET/CT-derived TA together with L1 SUV_avg_. Model performance was estimated in the paired diagnostic CT subset using 5-fold stratified cross-validation. Out-of-fold predicted probabilities were pooled across folds, and discrimination/calibration was summarized using the area under the receiver operating characteristic curve (AUC), average precision, and Brier score. In addition, diagnostic CT-L1 values between 110 and 160 HU were prespecified as the gray zone to evaluate whether ^18^F-NaF uptake could refine stratification when structural attenuation alone was indeterminate.

To preserve continuity with the prior DXA-anchored ^18^F-NaF study, the following SUVavg categories were carried forward for translational interpretation: <4.9 as low uptake, 4.9 to <5.7 as intermediate uptake, and ≥5.7 as higher uptake (18). These thresholds were likewise prespecified and carried over unchanged from that prior study; they should be regarded as exploratory.

### Metabolic-structural discordance analysis

Patient-level structural status was defined using mean PET/CT-derived TA across L1-L4, dichotomized at 135 HU (low TA <135 HU; higher TA ≥135 HU), and metabolic status was defined using mean SUV_avg_ across L1-L4, dichotomized at the previously derived ^18^F-NaF threshold of 4.9 (low uptake <4.9; higher uptake ≥4.9). These two binary dimensions were combined to generate four mutually exclusive phenotypes: higher TA plus higher uptake, higher TA plus low uptake, low TA plus higher uptake, and low TA plus low uptake.

### Statistical analysis

Statistical analyses were performed using Python-based statistical workflows. Normality was assessed with the Shapiro-Wilk test. Continuous variables are presented as mean ± standard deviation (SD) or median (interquartile range [IQR]), as appropriate, and categorical variables as counts and percentages.

To assess the agreement and feasibility of PET/CT-derived TA, Pearson correlation coefficients were calculated between PET/CT-derived L1 attenuation and stand-alone diagnostic CT-L1 and are reported with Fisher z-transformed 95% confidence intervals. Agreement was assessed using a two-way random-effects, absolute-agreement, single-measure intraclass correlation coefficient, with patient-level bootstrap 95% confidence intervals. Bland-Altman analysis was performed to estimate the mean difference and 95% limits of agreement between the two measurements.

Structural-metabolic association was evaluated at both patient and vertebral levels. At the patient level, the correlation between mean lumbar SUV_avg_ and mean PET/CT-derived TA across L1-L4 was assessed using Pearson correlation. At the vertebral level, same-level correlations were calculated for L1-L4. Age-adjusted linear models were then used to estimate the slope relating SUV_avg_ to TA at each vertebral level. To account for clustering of vertebrae within patients, a pooled mixed-effects model was used to estimate the change in SUV_avg_ per 10-HU increase in same-level TA.

To evaluate the incremental value of ^18^F-NaF metabolism for gray-zone stratification, cross-validated model performance was compared between low-dose CT-only and combined low-dose CT plus SUV models at the prespecified diagnostic CT-L1 thresholds. A two-sided p value <0.05 was considered statistically significant.

## Results

### Baseline characteristics

**Figure 2:**
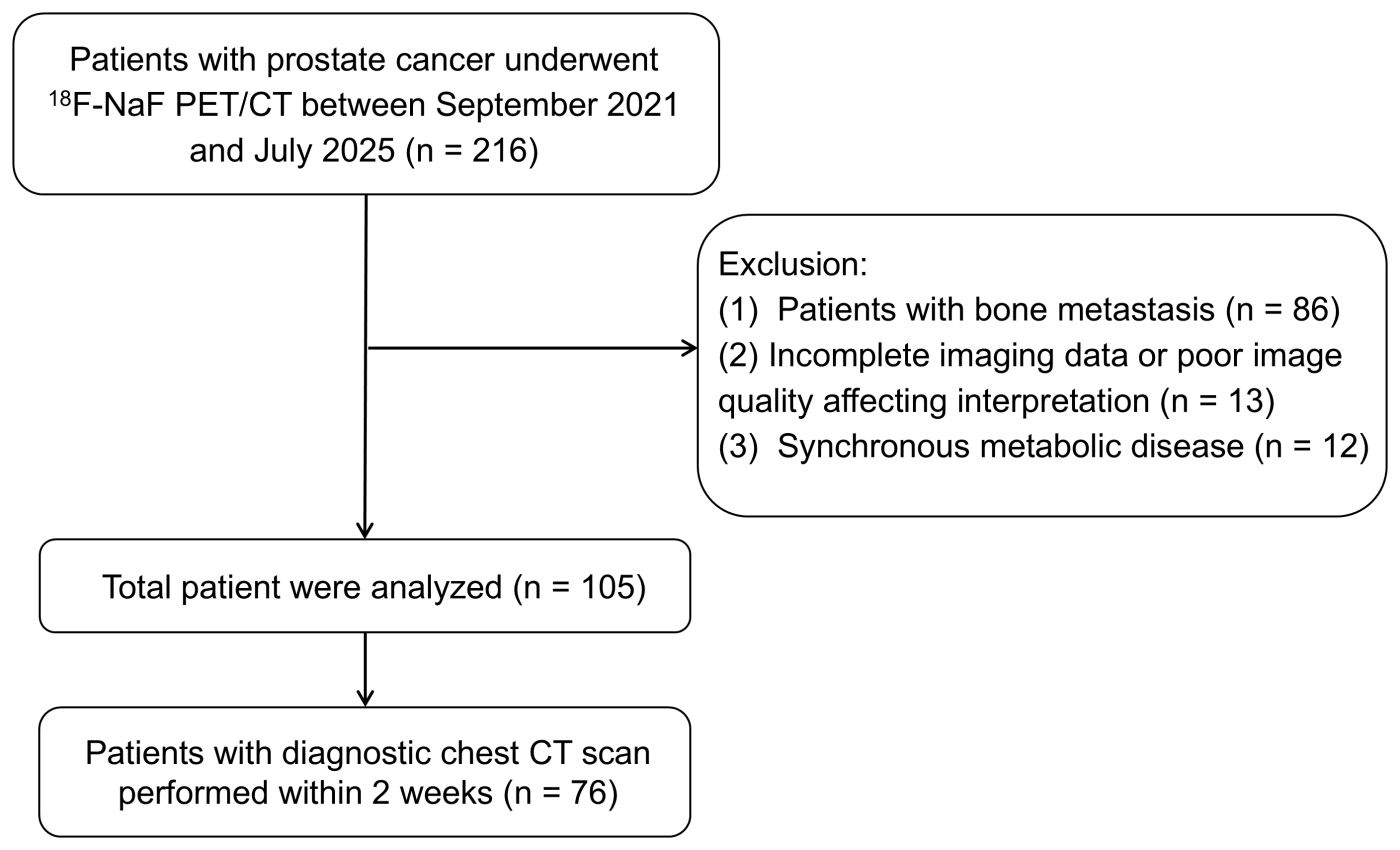
Study flowchart of the analytic cohort and prespecified subsets.

Between September 2021 and July 2025, 216 men with prostate cancer underwent ^18^F-NaF PET/CT at our institution. After exclusion of 86 patients with skeletal metastases, 13 with incomplete or suboptimal imaging data, and 12 with synchronous metabolic bone disease, the final analytic cohort comprised 105 men with non-metastatic prostate cancer (**Figure 2**). Mean age was 69.8 ± 7.8 years (median, 69.0 years). Ninety-five patients (90.4%) underwent ^18^F-NaF PET/CT for initial staging and 10 (9.6%) for follow-up. Current or former smokers accounted for 12 patients (11.4%). Median duration of previous treatment was 4.5 months (IQR, 3.0-5.8). A paired diagnostic subgroup of 76 patients (72.4%) also had a stand-alone diagnostic CT examination including L1 within two weeks of PET/CT.

### Feasibility of low-dose CT trabecular attenuation for opportunistic bone health screening

**Figure 3:**
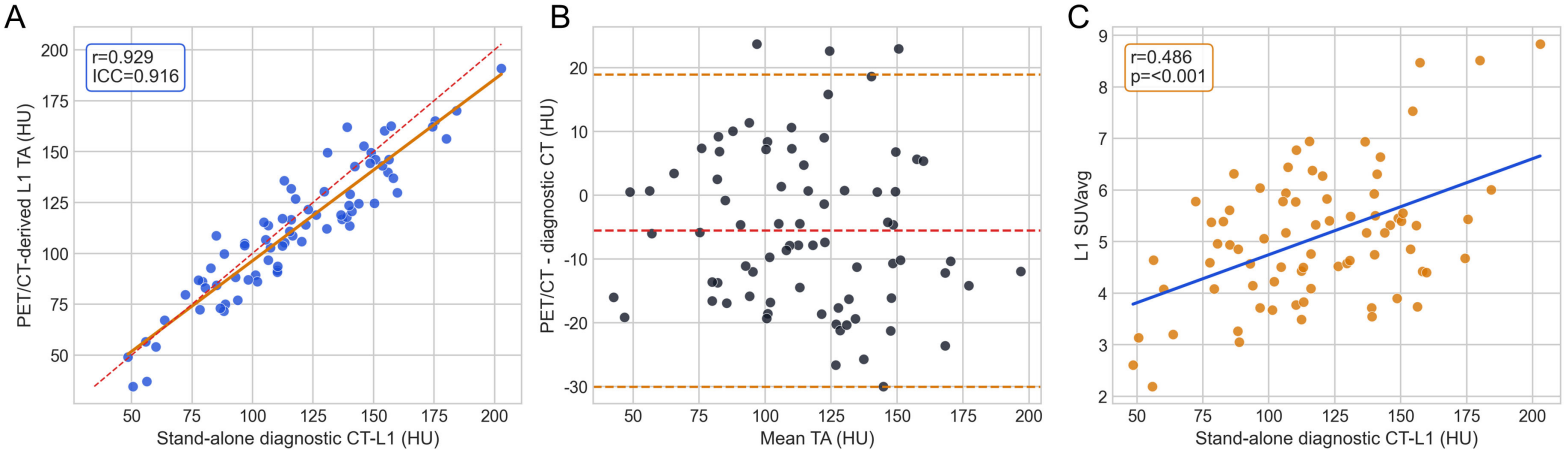
Structural and metabolic relationships with stand-alone diagnostic CT-L1. **(A)** Correlation between PET/CT-derived L1 trabecular attenuation and stand-alone diagnostic CT-L1. **(B)** Bland-Altman agreement for PET/CT-derived L1 trabecular attenuation versus stand-alone diagnostic CT-L1. **(C)** Correlation between L1 SUVavg and stand-alone diagnostic CT-L1.

In the paired subset of 76 patients, PET/CT-derived L1 TA correlated strongly with stand-alone diagnostic CT-L1 (r = 0.929; 95% CI, 0.890 to 0.955; p<0.001; **Figure 3A**). Agreement was also high using ICC (ICC = 0.916; 95% bootstrap CI, 0.877 to 0.941; **Figure 3B**). Although the diagnostic and low-dose CT acquisitions shared a 120-kV peak voltage, differences in dose and reconstruction can affect absolute HU; the small Bland-Altman mean difference (-5.55 HU; 95% limits of agreement -30.04 to 18.94 HU) indicates only minor systematic bias, supporting the practical transferability of literature-derived HU thresholds to our PET/CT-derived measurements while acknowledging residual measurement variability. Within the same paired subset, L1 SUV_avg_ also correlated positively with stand-alone diagnostic CT-L1, although less strongly than PET/CT-derived TA (r = 0.486; 95% CI, 0.292 to 0.641; p<0.001; **Figure 3C**). Together, these findings indicate that the low-dose CT component of ^18^F-NaF PET/CT can provide a practical structural layer for opportunistic lumbar bone health assessment, while the ^18^F-NaF metabolic signal shows a parallel but less tightly coupled relationship with the external structural reference.

### Incremental value of ^18^F-NaF metabolism for gray-zone stratification

**Figure 4:**
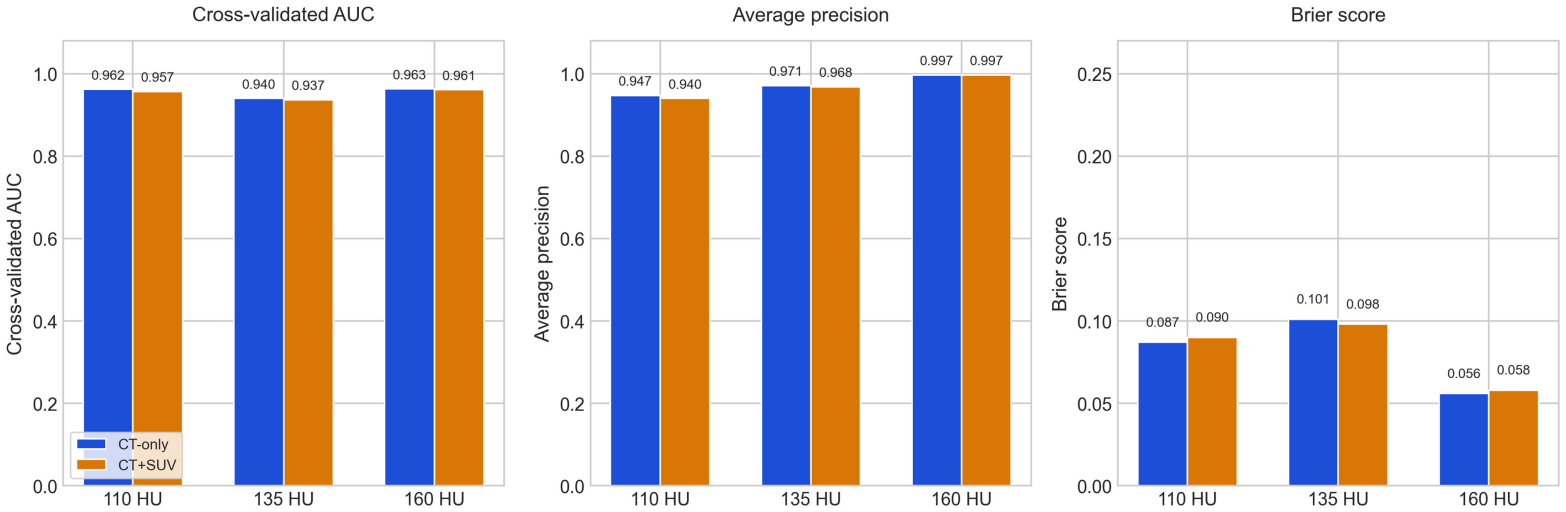
Cross-validated model comparison in the paired diagnostic CT subset, showing AUC, average precision, and Brier score for low-dose CT-only versus low-dose CT plus SUV models across diagnostic CT-L1 thresholds.

**Figure 5:**
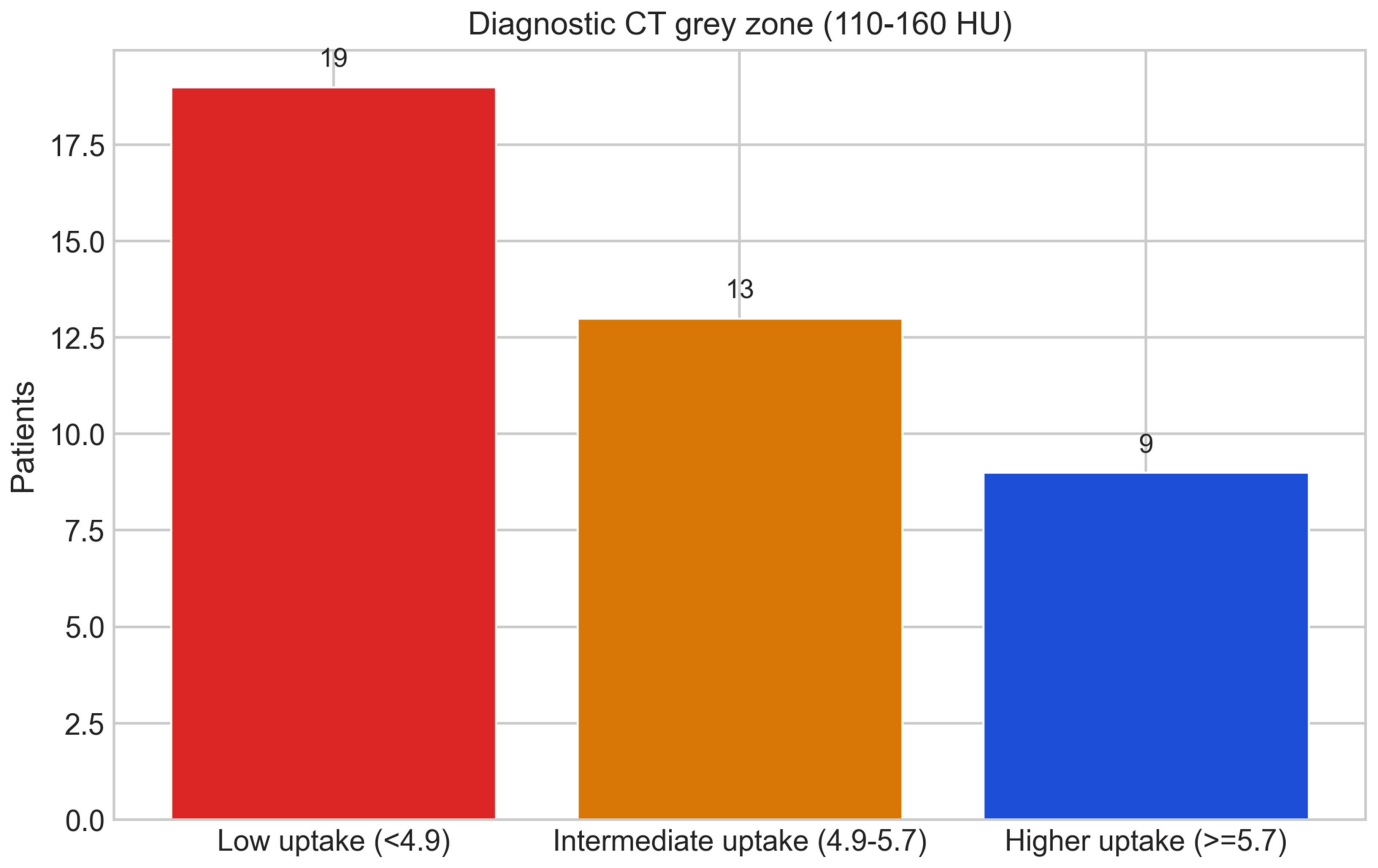
Metabolic stratification of patients within the stand-alone diagnostic CT gray zone (110–160 HU).

Cross-validated discrimination for low stand-alone diagnostic CT-L1 thresholds was evaluated in the 76-patient paired diagnostic CT subset. The number of events/non-events was 30/46 for diagnostic CT-L1 <110 HU, 49/27 for <135 HU, and 71/5 for <160 HU. At the 135-HU threshold, the AUC was 0.940 (95% CI, 0.888 to 0.982) for the low-dose CT-only model and 0.937 (95% CI, 0.882 to 0.981) for the low-dose CT plus SUV model; the corresponding average precision values were 0.971 (95% CI, 0.939 to 0.992) and 0.968 (95% CI, 0.937 to 0.991), and the Brier scores were 0.101 (95% CI, 0.059 to 0.150) and 0.098 (95% CI, 0.058 to 0.149). At the 110-HU threshold, performance again differed only minimally (AUC 0.962 [95% CI, 0.916 to 0.988] vs 0.957 [95% CI, 0.914 to 0.987]; average precision 0.947 [95% CI, 0.869 to 0.985] vs 0.940 [95% CI, 0.869 to 0.983]; Brier score 0.087 [95% CI, 0.048 to 0.135] vs 0.090 [95% CI, 0.049 to 0.137]). Similarly little separation was seen at the 160-HU threshold (AUC 0.963 [95% CI, 0.913 to 1.000] vs 0.961 [95% CI, 0.906 to 1.000]; average precision 0.997 [95% CI, 0.993 to 1.000] vs 0.997 [95% CI, 0.992 to 1.000]; Brier score 0.056 [95% CI, 0.014 to 0.102] vs 0.058 [95% CI, 0.016 to 0.105]) (**Figure 4**). Overall, this represents an important negative finding: adding SUV_avg_ did not materially improve whole-cohort discrimination when the reference outcome was itself attenuation based, given that much of the predictive information was already contained in the low-dose CT measure. However, the gray-zone analysis yielded an exploratory observation: among 41 patients with stand-alone diagnostic CT-L1 between 110 and 160 HU, 19 (46.3%) were classified as low uptake (**Figure 5**). This subgroup represents cases in which ^18^F-NaF metabolism might refine triage when structural attenuation alone is indeterminate, although its clinical significance cannot be established without DXA, fracture, or follow-up data.

### Association between ^18^F-NaF uptake and structural trabecular attenuation

**Figure 6:**
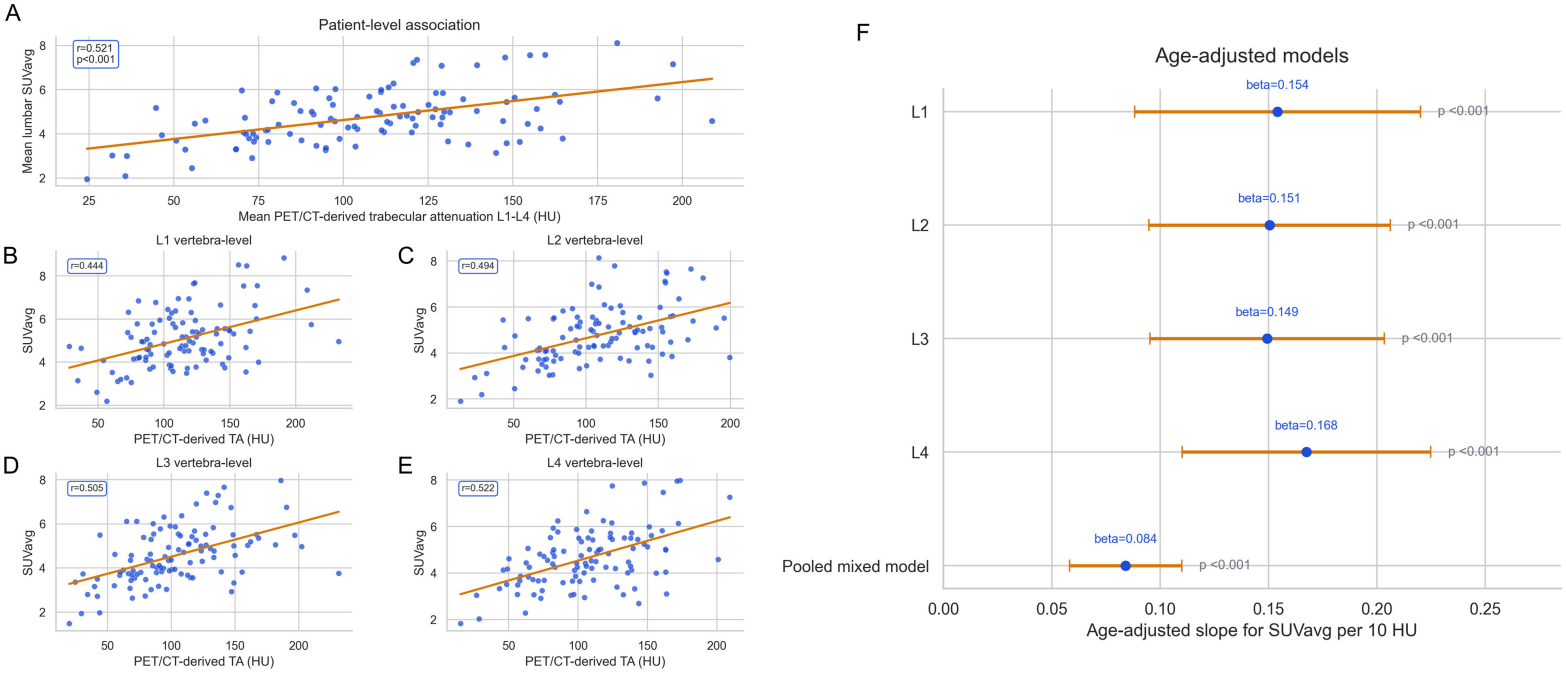
Summary of metabolic-structural association analyses. **(A)** Patient-level correlation between mean lumbar SUV_avg_ and mean PET/CT-derived trabecular attenuation across L1-L4. **(B–E)** Vertebra-level same-level correlations for L1-L4. **(F)** Age-adjusted beta estimates including the pooled mixed-effects model.

Among the 105 patients who underwent ^18^F-NaF PET/CT, mean SUV_avg_ across L1-L4 correlated positively with mean PET/CT-derived TA across L1-L4 (r = 0.521; 95% CI, 0.366 to 0.648; p<0.001; **Figure 6A**). Vertebral analyses showed consistent positive same-level relationships across all lumbar levels, with correlation coefficients ranging from 0.444 at L1 (95% CI, 0.274 to 0.587) to 0.522 at L4 (95% CI, 0.367 to 0.650) (**Figures 6B–E**). In age-adjusted linear models, the slope relating SUVavg to TA remained positive at every vertebral level: 0.154 per 10 HU at L1 (95% CI, 0.089 to 0.219), 0.151 at L2 (95% CI, 0.095 to 0.206), 0.149 at L3 (95% CI, 0.096 to 0.203), and 0.168 at L4 (95% CI, 0.111 to 0.224). In the vertebra-level mixed-effects model, each 10-HU increase in same-level TA was associated with a 0.084-unit increase in SUVavg (95% CI: 0.058 to 0.110; p < 0.001; **Figure 6F**). These results recapitulate the association between structural and metabolic pattern, as well as support the interpretation that lower lumbar ^18^F-NaF uptake may track poorer underlying bone status.

### Metabolic-structural discordance for all patients

**Figure 7:**
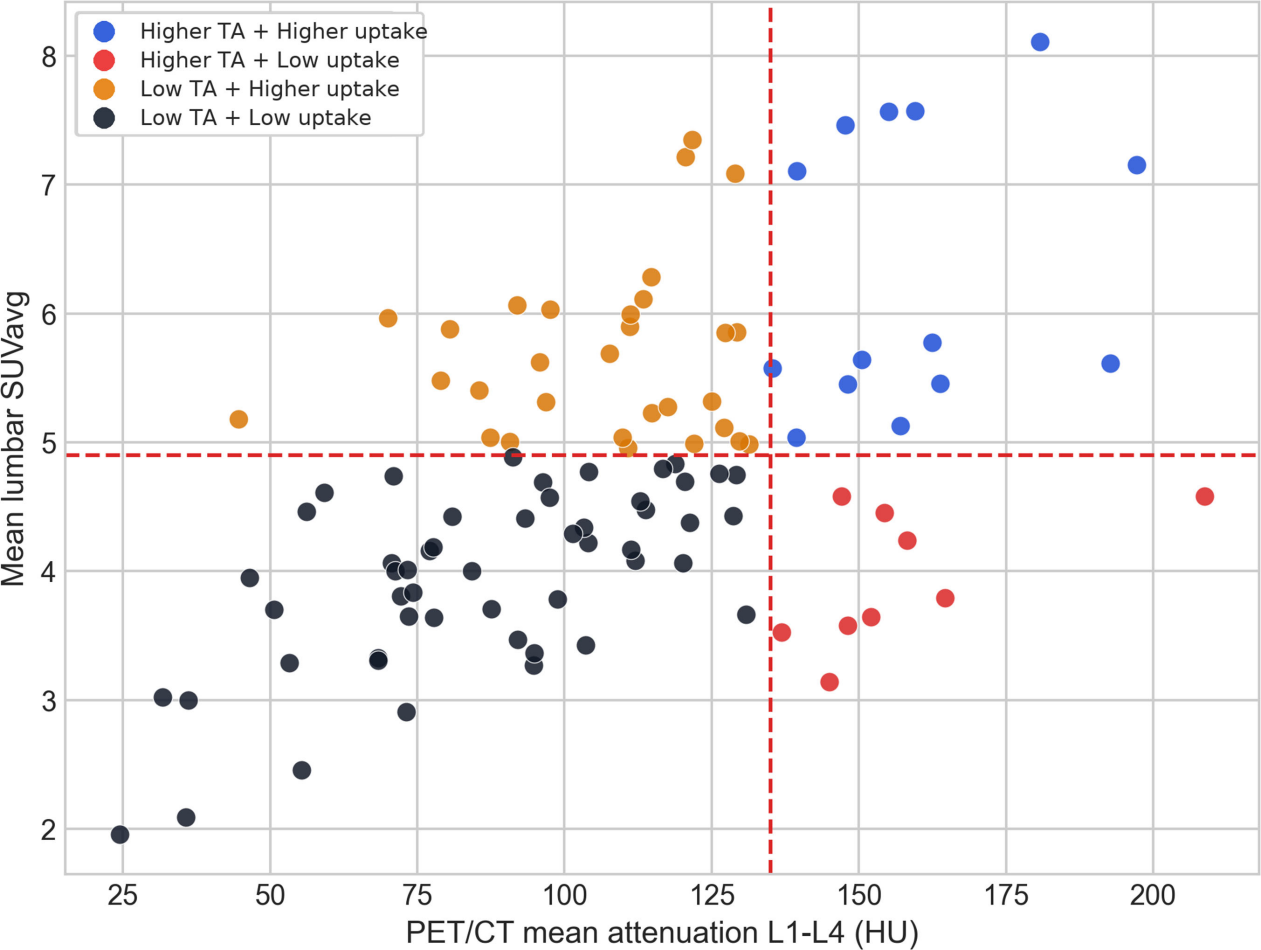
Dual-parameter phenotype distribution based on mean PET/CT-derived trabecular attenuation and mean lumbar SUV_avg_.

**Figure 8:**
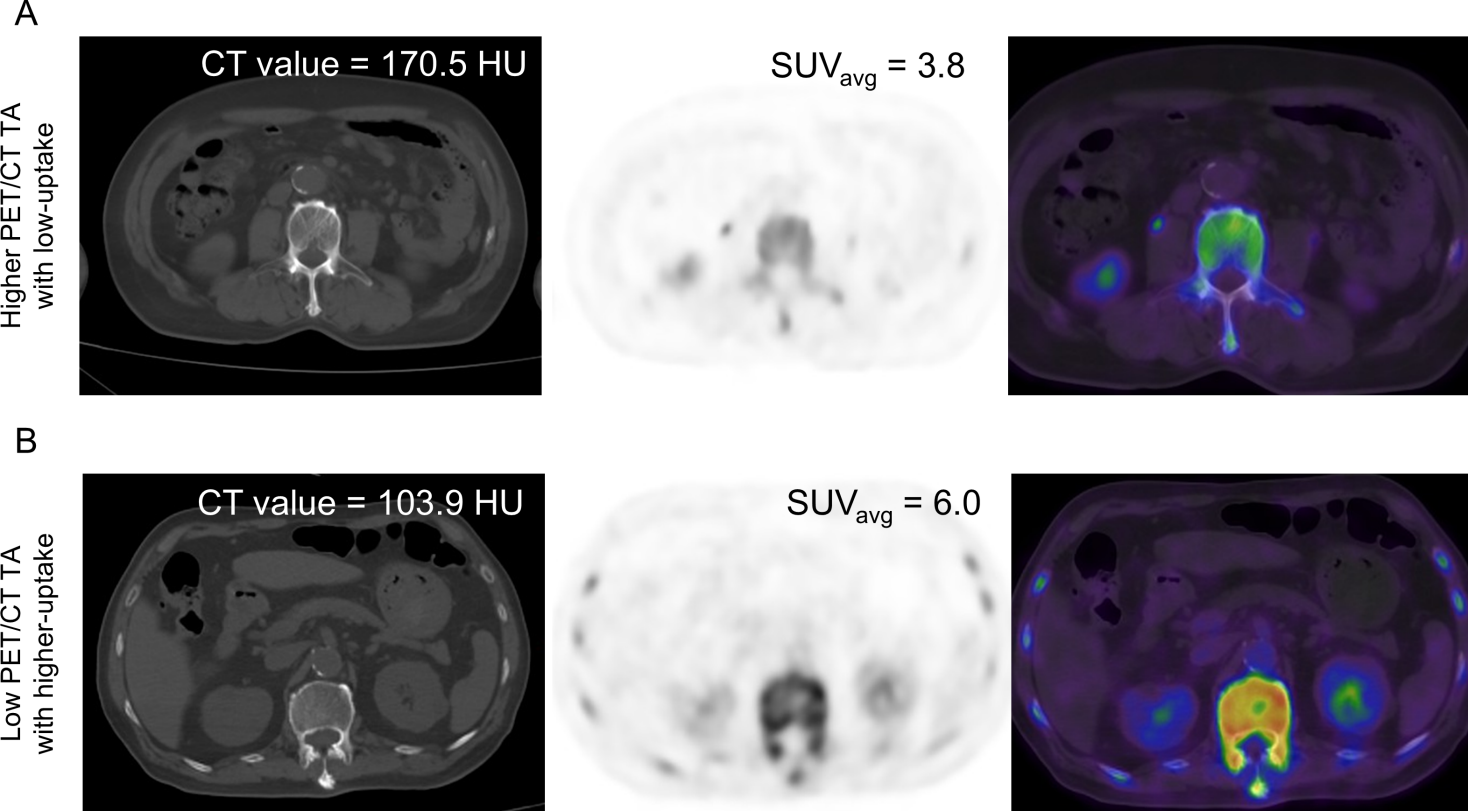
Example of metabolic-structural discordance. **(A)** Higher PET/CT TA but low-uptake phenotype. **(B)** Low PET/CT TA but higher-uptake phenotype.

One of the key translational observations was the presence of a higher-PET/CT-TA but low-uptake phenotype in 9 of 105 patients (**Figures 7**, **8A**). This group would not be highlighted by low-dose CT TA-only screening, yet its metabolic profile may indicate poorer bone status. Conversely, 30 patients showed low PET/CT TA with higher uptake, suggesting that metabolic and structural measures do not always decline synchronously (**Figure 8B**). Taken together, these patterns support the concept that ^18^F-NaF PET/CT is not merely a low-dose CT TA platform with an added tracer, but rather a combined structural and metabolic assessment of lumbar bone health.

## Discussion

This study extends our previous DXA-anchored lumbar ^18^F-NaF work into a clinically relevant prostate cancer setting and supports the interpretation of ^18^F-NaF PET/CT as a dual-parameter bone health examination. ^18^ The low-dose CT component proved structurally feasible for opportunistic lumbar assessment, whereas the metabolic contribution of ^18^F-NaF uptake emerged most clearly in gray-zone patients in whom structural attenuation alone was indeterminate. Lumbar ^18^F-NaF uptake remained positively associated with trabecular attenuation at both patient and vertebral levels, recapitulating the biologic pattern seen in our prior DXA-based study. Finally, metabolic-structural discordance phenotypes identified within the same examination suggest that bone structure and bone metabolism do not invariably decline in parallel.

The structural feasibility result provides the foundation for any opportunistic bone health framework embedded in ^18^F-NaF PET/CT. The strong agreement between PET/CT-derived L1 TA and stand-alone diagnostic CT-L1 (r = 0.929, ICC = 0.916) indicates that the low-dose CT component is not merely a technical adjunct for attenuation correction, but a usable structural layer for lumbar bone assessment. This magnitude of agreement is consistent with the broader opportunistic CT literature, in which L1 TA has been validated as a practical surrogate of bone mineral density across routine clinical settings (7–11, 14). It also aligns with prior prostate cancer PET/CT studies using other tracers. Dauchez et al. (13) showed that CT attenuation from ^18^F-fluorocholine PET/CT supports opportunistic osteoporosis and vertebral fracture screening in prostate cancer, while Schwaiger et al. (12) demonstrated that vertebral and femoral bone density can be estimated from phantomless PET/CT. This tracer-specific behavior is further illustrated by PSMA-targeted imaging: Ninatti et al. (21) reported that lower bone density was associated with increased unspecific ^18^F-PSMA-1007 skeletal uptake, a relationship opposite in direction to the bone-specific ^18^F-NaF signal observed here. The present study instead used ^18^F-NaF PET/CT, whose tracer directly reflects skeletal perfusion, hydroxyapatite binding, and osteoblastic remodeling, thereby extending the concept from a primarily structural CT-attenuation framework to a dual structural-metabolic one. Thus, our analysis extends the PET/CT opportunistic screening concept from a primarily structural CT-attenuation framework to a dual structural-metabolic framework. At the same time, because the reference outcome in this study was CT-defined, our results should be interpreted as feasibility and hypothesis generation rather than clinical validation.

The more clinically relevant question is where the ^18^F-NaF metabolic signal adds value. When the reference outcome was defined by stand-alone diagnostic CT attenuation, adding SUV to low-dose CT-derived TA did not materially improve whole-cohort discrimination; AUC, average precision, and Brier score remained very similar across all prespecified thresholds. This was methodologically unsurprising because the target outcome was itself a CT-based structural measure, meaning that much of the predictive information was already captured by the low-dose CT component. The more informative finding emerged in the gray zone. Among patients with stand-alone diagnostic CT-L1 values between 110 and 160 HU, nearly half were classified as low uptake. This suggests that ^18^F-NaF metabolism may help refine clinical triage when structural attenuation alone is equivocal, rather than materially changing whole-cohort prediction of a CT-defined endpoint.

The biologic plausibility of this interpretation is reinforced by the observed association between ^18^F-NaF uptake and TA. Mean lumbar SUV_avg_ correlated positively with mean lumbar TA at the patient level, and the same pattern was preserved across individual vertebrae and in age-adjusted models. In the pooled mixed-effects model, each 10-HU increase in same-level TA was associated with a 0.084-unit increase in SUV_avg_. These results are consistent with our prior DXA-anchored study, in which lower lumbar ^18^F-NaF uptake was linked to lower bone mineral density (18). They are also congruent with work by Chesnais et al., who reported that lower vertebral ^18^F-NaF uptake was associated with higher fracture risk in prostate cancer (22). Together, these data are consistent with the interpretation that lower lumbar ^18^F-NaF uptake may reflect an unfavorable bone phenotype rather than random tracer variation, though this remains to be confirmed against independent clinical endpoints.

One notable observation in this study was the identification of metabolic-structural discordance phenotypes. As an exploratory observation, nine patients exhibited higher PET/CT-derived TA but low uptake. This phenotype would not be flagged by low-dose CT TA alone, yet the metabolic profile is directionally consistent with poorer bone status. A plausible interpretation is that reduced ^18^F-NaF uptake may capture an earlier disturbance in bone remodeling before structural loss becomes sufficiently pronounced to lower TA. Conversely, 30 patients showed low TA with higher uptake, suggesting that structural compromise may coexist with relatively preserved or compensatory metabolic activity. Regardless of the underlying mechanism, these discordant patterns underscore that structural attenuation and ^18^F-NaF uptake are related but not interchangeable. This is central to the translational message of the study: ^18^F-NaF PET/CT should not be viewed simply as a low-dose CT attenuation platform with an added tracer, but as a combined structural and metabolic examination of lumbar bone health.

From a clinical standpoint, this dual-parameter framework is best regarded as an opportunistic, hypothesis-generating risk-enrichment approach rather than a replacement for DXA or a new diagnostic standard for osteoporosis. In practice, routine ^18^F-NaF PET/CT examinations performed for staging or surveillance could be used to extract lumbar TA and SUV_avg_, thereby flagging patients with low TA, those in the structural gray zone who also have low uptake, and those with a higher-TA/low-uptake discordance phenotype. Such patients could then be referred for formal DXA, dedicated bone health assessment, or treatment consideration under existing guidance (1–4).

Several limitations should be acknowledged. This was a retrospective single-center study, which limits generalizability. Stand-alone diagnostic CT was available only in a subset of patients, and systematic DXA and vertebral fracture assessment were not available; consequently, we could not perform formal diagnostic accuracy analyses against established osteoporosis endpoints. Several determinants of bone density and ^18^F-NaF uptake, including androgen deprivation therapy exposure and duration, antiresorptive therapy, calcium and vitamin D supplementation, corticosteroid use, prior fragility fractures, body mass index, renal function, serum calcium/phosphate/alkaline phosphatase, PSA, tumor stage, and other comorbidities, were not systematically recorded in the source records and could not be reliably retrieved. Consequently, these variables could not be incorporated into the baseline characteristics or used for confounder adjustment or treatment-stratified analyses. The cross-sectional design also precludes inference regarding longitudinal bone loss or future fracture risk. Finally, the dual-parameter framework proposed here has not yet been externally validated or tested prospectively. Even so, the study provides a coherent translational bridge from our prior mechanistic ^18^F-NaF observation to a clinically relevant use case in prostate cancer.

## Conclusion

^18^F-NaF PET/CT may serve dual functions in prostate cancer including oncologic skeletal assessment and opportunistic bone health evaluation. The low-dose CT component provides structurally reliable lumbar TA measurements, whereas ^18^F-NaF uptake contributes complementary metabolic information that may refine gray-zone stratification and reveal metabolic-structural discordance. These exploratory findings support the concept of ^18^F-NaF PET/CT as a multifunctional examination; however, diagnostic accuracy against established osteoporosis or fracture endpoints was not assessed, and prospective validation against DXA and fracture endpoints remains required before routine clinical implementation.

## Data Availability

The raw data supporting the conclusions of this article will be made available by the authors, without undue reservation.
